# Starch Extraction from Rejected *Solanum tuberosum* L. Optimized by Box–Behnken Design: Enzymatic and UV-Vis Spectrophotometric Quantification

**DOI:** 10.3390/ma19153197

**Published:** 2026-07-27

**Authors:** Leidy Valentina Olaya Bocanegra, Karoll Stefany Gómez Triana, Diana Angélica Varela-Martínez, Lady Dayana Barajas Gómez

**Affiliations:** 1 Chemical Engineering Program, Faculty of Engineering, Universidad EAN, Calle 79 n° 11-45, Bogotá 110221, Colombia; lolayab61588@universidadean.edu.co (L.V.O.B.); kgomezt77901@universidadean.edu.co (K.S.G.T.); 2 Department of Basic Sciences, Faculty of Engineering, Universidad EAN, Calle 79 n° 11-45, Bogotá 110221, Colombia; 3 Comestibles Ricos S.A. (Super Ricas), Bogotá 110121, Colombia; lady.barajas@superricas.com

**Keywords:** potato starch, agro-industrial waste, Box–Behnken design, response surface methodology, enzymatic quantification, circular economy, biopolymer, *Solanum tuberosum* L., Colombia

## Abstract

**Highlights:**

- Box–Behnken RSM identified temperature and solid:liquid ratio as dominant drivers of starch extraction from rejected Pastusa potato.
- Rejected tubers yielded 5.41 ± 1.30% starch—1.9× higher than commercial-grade material under identical optimal conditions.
- Intra-extraction CV < 1.5%; relative standard deviation between independent extractions < 5%.
- UV-Vis iodine–starch complex (600 nm; R^2^ = 0.9830) confirmed the characteristic colorimetric response of native starch, consistent with amylose-rich composition.
- Rejected agro-industrial potato constitutes a viable circular-economy feedstock for native starch recovery.

**Abstract:**

Industrial potato processing generates substantial rejected-tuber streams that are rarely valorized. This study optimized wet-process starch extraction from rejected *Solanum tuberosum* L. (Pastusa variety) sourced from a Colombian snack processor (Comestibles Ricos S.A., Super Ricas, Bogotá), using a Box–Behnken design with three factors: temperature (20–40 °C), solid-to-liquid ratio (1:2–1:4, *w*/*v*), and citric acid concentration (0–6%, *w*/*v*; n = 26 per material). The reduced model (F = 9.236, *p* = 0.0003) identified temperature (β_1_ = +0.729) and solid-to-liquid ratio (β_2_ = +0.695) as the dominant yield drivers, with the identified optimum located at the boundary of the tested domain, indicating that an expanded range may yield further gains. Across the full experimental domain, rejected-grade potato produced significantly higher starch yields than standard-grade material (3.06 ± 1.20% vs. 1.85 ± 0.53%; Welch’s t = 4.70, *p* < 0.0001, Cohen’s d = 1.30). Under the identified optimal conditions (40 °C, 1:4 *w*/*v*, 3% citric acid), rejected-grade tubers yielded 5.41 ± 1.30%, 3.6-fold higher than standard-grade material processed under the same conditions (1.49 ± 0.14%), consistent with pre-existing intracellular starch release from mechanically damaged tubers. Total starch content reached 83.4 ± 2.6% (dry basis) as determined by the Megazyme K-TSTA-100A enzymatic kit (AOAC 996.11), with CV < 1.5% between independent extractions. UV-Vis spectrophotometry of the iodine–starch complex (600 nm; R^2^ = 0.9830) confirmed native amylose-rich starch identity. These results establish rejected Pastusa potato as a statistically robust circular-economy feedstock for native starch recovery.

## 1. Introduction

The potato (*Solanum tuberosum* L.) ranks among the four most globally consumed staple crops, with 376 million metric tonnes produced worldwide in 2021 [1]. Industrial processing—snack manufacturing, frozen products, and starch refining—generates substantial by-product streams that rarely receive systematic valorization [2]. Off-spec tubers rejected during quality-control grading due to irregular morphology, substandard diameter, or mechanical surface damage constitute a particularly underexplored residue; post-harvest potato losses are estimated to range between 15% and 35% across different regions and processing systems [3], and potato-processing waste alone is projected to reach 8000 kilotonnes per year by 2030, which is associated with greenhouse gas emissions equivalent to 5 Mt CO_2_ [1].

Potato starch, composed of amylose (18–23%) and amylopectin (77–82%), is one of the most commercially relevant biopolymers for food, pharmaceutical, packaging, adhesive, and textile applications [4,5]. Its granular structure, high swelling capacity, and film-forming properties make it an attractive matrix for biodegradable packaging materials, increasingly demanded in the context of regulatory restrictions on single-use plastics [6,7]. The starch content of *S. tuberosum* L. tubers ranges from 12 to 22% on a fresh-weight basis, depending on variety, growing region, harvest maturity, and storage conditions [8]. In the Andean highlands of Colombia, the Pastusa variety represents the primary raw material for domestic snack processors such as Super Ricas (Comestibles Ricos S.A., Bogotá). Despite its agronomic relevance, no published study has systematically characterized the starch recovery potential of off-spec Pastusa-variety tubers under optimized process conditions.

Wet-process starch extraction involves, in sequential unit operations, size reduction, soaking, filtration, pH adjustment, centrifugation, and drying, and its efficiency depends on temperature, dilution ratio, and pretreatment chemistry [9]. Citric acid is commonly employed to inhibit enzymatic browning and disrupt protein–starch surface associations, but its net effect on starch yield is non-linear and depends on interactions with temperature and dilution [10]. The Box–Behnken design (BBD), proposed by Box and Behnken (1960) [11], permits efficient estimation of second-order polynomial response surfaces with fewer experimental runs than a full central composite design, and has been applied to optimize starch extraction from cassava, sweet potato, and banana [12]. Its application to rejected Andean potato represents a gap that this work addresses.

Starch quantification in complex plant matrices requires analytically robust methods. The enzymatic approach based on thermostable α-amylase and amyloglucosidase (AMG), detected with the glucose oxidase–peroxidase (GOPOD) reagent, constitutes the internationally validated reference standard (AOAC Official Method 996.11; AACC Method 76-13.01; ICC Method 168) and is implemented in the Megazyme K-TSTA-100A kit [13]. UV-Vis spectrophotometry of the iodine–starch complex provides a complementary, cost-accessible measurement sensitive to helical amylose fractions; together, both methods provide convergent analytical evidence for the identity and quality of the extracted starch fraction [14,15].

The present study reports the following: (i) optimization of starch extraction from rejected *S. tuberosum* L. by BBD-RSM; (ii) a systematic yield comparison between rejected-grade and commercial-grade tubers; (iii) total starch quantification by enzymatic kit (Megazyme AOAC 996.11) and UV-Vis spectrophotometric confirmation of starch identity; and (iv) contextualization within a circular economy framework for agro-industrial starch valorization.

## 2. Materials and Methods

### 2.1. Raw Material and Reagents

Rejected-grade *Solanum tuberosum* L. (Pastusa variety) tubers were supplied by Comestibles Ricos S.A. (Super Ricas, Bogotá, Colombia), a snack-processing company generating approximately 300–500 kg day^−1^ of off-spec material, as classified by substandard diameter (<35 mm) or surface defects incompatible with industrial slicing. Standard-grade tubers of the same variety, purchased at the Corabastos wholesale market (Bogotá, Colombia), served as the reference material. All tubers were processed within 48 h of reception and stored at 4 °C. Citric acid monohydrate (≥99.5%), NaOH pellets (≥97%), and commercial soluble starch standard were supplied by Merck KGaA (Darmstadt, Germany). Distilled water (conductivity < 2 µS cm^−1^) was used throughout.

### 2.2. Starch Extraction Protocol

Tubers were washed under running tap water, peeled manually, and grated to reduce particle size and maximize cell disruption. Approximately 100 g of grated potato was immersed in citric acid solution (0%, 3%, or 6%, *w*/*v*), prepared with distilled water pre-conditioned to the target extraction temperature (20, 30, or 40 °C), for 120 min under continuous gentle stirring to inhibit enzymatic browning, consistent with reports establishing citric acid as an effective anti-browning agent in Colombian potato varieties including Pastusa [16], and to facilitate protein–starch dissociation. Soaking time was fixed at 120 min based on established practice for enzymatic browning inhibition and cell-wall softening in cut potato tissue, where immersion periods of 1 to 2 h are commonly sufficient to achieve stable polyphenol oxidase inhibition without inducing excessive microbial growth or starch hydrolysis at an ambient temperature; this parameter was not included as a design factor since a preliminary literature review identified temperature, solid-to-liquid ratio, and citric acid concentration as the primary determinants of wet-process starch extraction yield. The mixture was filtered through double-layer fine-mesh polypropylene cloth; the solid retentate was discarded. Distilled water pre-conditioned to the same target temperature was added to the filtrate according to the solid-to-liquid ratio assigned to each experimental run (1:2, 1:3, or 1:4, *w*/*v*), and the suspension was homogenized at high speed for 60 s (Osterizer Classic, Sunbeam Products Inc., Boca Raton, FL, USA). A second filtration step removed residual fibrous material, and the pH was adjusted to 5.0 ± 0.1 with 1 M NaOH or citric acid (HI98190, Hanna Instruments, Smithfield, RI, USA). The suspension was centrifuged at 2500 rpm (769× *g*; 11.0 cm rotor radius) for 15 min (Hermle Z206A, Hermle Labortechnik GmbH, Wehingen, Germany, Germany). The supernatant was decanted, and the sediment dried at 45 °C for 24–26 h until constant mass, ground in an analytical mill (IKA A11, IKA-Werke GmbH & Co. KG, Staufen im Breisgau, Germany), and stored in sealed polyethylene bags until analysis. Extraction yield was calculated as: Y (%) = (m_starch,dry/m_fresh sample) × 100.

### 2.3. Box–Behnken Experimental Design and Statistical Analysis

A three-factor Box–Behnken design (BBD) was applied independently to rejected-grade and standard-grade potato. The independent variables and their coded levels are given in Table 1. The design generated 13 unique treatment combinations (12 midpoint-edge points + 1 center point), each replicated in biological duplicate (n = 2 independent extractions per condition, 26 observations per material), consistent with standard practice for resource-intensive wet-chemistry optimization studies. The response variable was starch extraction yield (% *w*/*w*, dry basis). A full second-order polynomial model was initially fitted, and backward elimination at α = 0.05 was applied to obtain a reduced model. Statistical analysis was performed with JMP Pro 17.0 (SAS Institute Inc., Cary, NC, USA), including global F-test, Lack of Fit (LoF), and regression diagnostics (R^2^, R^2^adj, R^2^pred, CV%). A second-order polynomial model was fitted rather than a first-order model because response surface methodology requires a quadratic term to characterize curvature and locate a stationary point, and because a linear model cannot estimate the two-factor interaction terms that the full model identified as significant, consistent with standard practice for Box–Behnken designs.

### 2.4. Moisture Determination

Moisture content was determined gravimetrically by drying approximately 1 g of starch powder at 105 °C for 24 h (AOAC 930.15). Triplicate measurements were performed on each sample, and results were used to convert all starch determinations to a dry-weight basis.

### 2.5. Total Starch Quantification: Megazyme K-TSTA-100A (AOAC 996.11)

Total starch was determined using the Megazyme Total Starch (AA/AMG) Assay Kit (K-TSTA-100A, Neogen-Megazyme, Bray, Ireland), following the Rapid Total Starch (RTS) procedure validated as AOAC Official Method 996.11 and AACC International Method 76-13.01. Approximately 100 mg of starch powder (previously dried at 105 °C for 24 h to constant mass) was weighed in triplicate alongside a sample blank. Samples were suspended in Buffer A containing thermostable α-amylase and incubated at 100 °C for 15 min with vortex agitation to gelatinize and partially hydrolyze the starch. After cooling, amyloglucosidase (AMG, *Aspergillus niger*) was added for saccharification at 50 °C for 30 min, quantitatively converting dextrins to free glucose. Tubes were centrifuged at 13,000 rpm for 5 min; 1.0 mL of supernatant was diluted with 10 mL of Buffer A (1:11 dilution), and 1.0 mL of this dilution was further diluted with 1.0 mL of Buffer A (1:2 dilution), yielding a composite dilution factor D = 22. A 0.1 mL aliquot was incubated with GOPOD reagent at 50 °C for 20 min, and absorbance was measured at 510 nm (Shimadzu UV-1900i, Shimadzu Corp., Kyoto, Japan). A glucose control standard (0.5 mg mL^−1^) was included in each analytical batch. Total starch on a wet-weight basis was calculated as: % Starch (w.b.) = (ΔA × F × 10.2 × D × 0.90)/W, where ΔA = A_sample_ − A_blank_; F = 50/A_510_ (glucose control); 10.2 mL is the extraction volume; D = 22 is the composite dilution factor; W is sample mass (mg); and 0.90 is the anhydrocorrection factor (162/180). Dry-basis content was calculated from wet-basis values and gravimetric moisture data. All determinations were performed in triplicate on two independent extractions.

### 2.6. UV-Vis Spectrophotometry: Iodine–Starch Complex Method

Iodine-reactive starch was assessed by UV-Vis spectrophotometry based on the formation of the amylose–triiodide inclusion complex [14]. A Lugol’s reagent (0.2% I_2_/2.0% KI, *w*/*v*) was prepared by dissolving KI in distilled water prior to adding I_2_, and stored at 4 °C in amber glass. A sodium acetate buffer (0.2 M, pH 4.5 ± 0.05) was used as the reaction medium to stabilize complex formation and minimize amylopectin interference [15].

For sample preparation, approximately 50 mg of starch powder was accurately weighed into a 50 mL screw-cap centrifuge tube. One milliliter of ethanol (95%, *v*/*v*) was added and the suspension was vortexed for 30 s to wet the powder and prevent clumping. Nine milliliters of 1.0 M NaOH were then added, the tube was vortexed for 15 s and placed in a boiling water bath (100 °C) for 15 min with manual agitation every 5 min until a translucent solution free of visible particles was obtained. After cooling to ambient temperature, the pH was adjusted to 4.5 ± 0.1 with 1.0 M HCl, monitored with a calibrated glass electrode. The solution was quantitatively transferred to a 100 mL volumetric flask and brought to volume with distilled water, yielding a nominal stock concentration of 500 µg mL^−1^.

A calibration curve was constructed from commercial soluble starch (Merck KGaA, Darmstadt, Germany) standards at 0, 25, 50, 100, 150, 200, and 250 µg mL^−1^ prepared in acetate buffer. For each standard and sample, 2.0 mL of solution was transferred to a glass tube, mixed with 0.2 mL of Lugol’s reagent and 2.8 mL of acetate buffer (total reaction volume 5.0 mL), and incubated in the dark at ambient temperature (20–25 °C) for exactly 10 min; absorbance was then measured at 600 nm against a reagent blank (Shimadzu UV-1900i, Shimadzu Corp., Kyoto, Japan). All measurements were performed in duplicate (raw calibration data provided in Table S2).

### 2.7. Generative AI Disclosure

During the preparation of this manuscript, the authors used Claude (Anthropic, claude.ai) for scientific writing assistance and language editing. The authors have reviewed and edited all output and take full responsibility for the content of this publication. No AI tool was used for data generation, experimental design, statistical analysis, or interpretation of results.

## 3. Results and Discussion

### 3.1. Box–Behnken Design: Global Model Validation and ANOVA

The 26-observation dataset for rejected-grade potato yielded a mean extraction yield of 3.056 ± 1.196% (SD), with values spanning 0.967–6.324% and a coefficient of variation of 39.1%. Shapiro–Wilk normality testing performed on the residuals of the reduced model (W = 0.9558; *p* = 0.3151) confirmed a normally distributed residual structure, as required for ordinary least-squares regression in RSM [11]. The full second-order model was globally significant (F = 4.809; *p* = 0.0032). Backward elimination at α = 0.05 yielded a reduced model retaining four terms beyond the intercept (Table 2), which was highly significant (F = 9.236; *p* = 0.0003). The Lack of Fit test, computed from the true replicate structure of the design (13 unique conditions, pure error df = 13), was non-significant (F = 2.295; *p* = 0.0882). Myers et al. establish that the primary validity criterion in RSM is the global F-test together with a non-significant Lack of Fit, both of which are satisfied here. Regression diagnostics (R^2^ = 0.634; R^2^adj = 0.564; R^2^pred = 0.449; CV = 25.8%) are consistent with extraction studies on biological matrices, where CV values of 18–27% are routinely reported.

The predictive capacity of the reduced model warrants explicit discussion. While the global F-test confirms that the model captures real structure in the data (F = 9.236, *p* = 0.0003), the predicted R^2^ obtained by leave-one-out cross-validation (R^2^pred = 0.449) indicates that the model explains less than half of the variability in new observations. This is a material limitation for a model used to identify a process optimum, and it should not be dismissed on the basis of the global F-test alone. Furthermore, the identified optimum (40 °C, 1:4 *w*/*v*, 3% citric acid) lies at the +1 coded level for both temperature and solid-to-liquid ratio, placing it at the boundary of the tested experimental domain. An optimum located at the edge of the design space is conventionally interpreted as evidence that the true optimum may lie outside the range explored, rather than as confirmation of a validated global maximum. Consequently, the conditions reported here should be understood as the optimum identified within the tested experimental domain, not as a validated global optimum. A follow-up design extending the upper bounds of temperature and solid-to-liquid ratio is recommended before these conditions are scaled to pilot or industrial operation.

The final reduced model in coded variables is given by Equation (1):

Ŷ = 2.697 + 0.729x_1_ + 0.695x_2_ + 0.664x_1_x_3_ − 0.605x_2_x_3_ (1)

with R^2^ = 0.634 and CV = 25.8%.

### 3.2. Effect of Process Variables on Starch Extraction Yield

**Figure 1:**
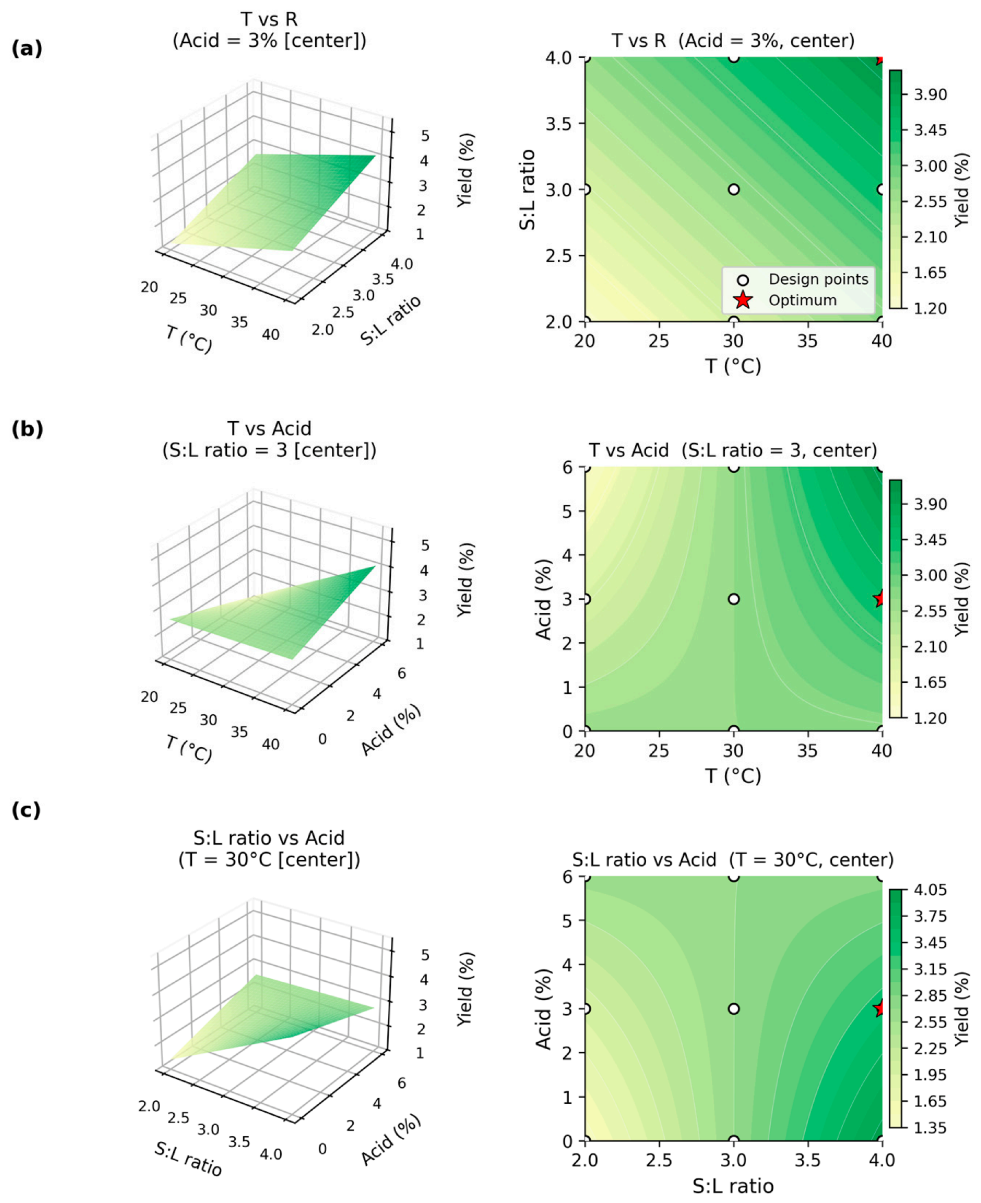
3D response surface plots (left) and contour plots (right) generated from the reduced Box–Behnken model described in Section 3.1, for starch extraction yield (%) from rejected *Solanum tuberosum* L.: (**a**) temperature vs. solid:liquid ratio at citric acid = 3% (center); (**b**) temperature vs. citric acid concentration at S:L ratio = 3 (center); (**c**) S:L ratio vs. citric acid concentration at T = 30 °C (center). White circles mark the projected positions of all 13 unique experimental design points. Red star (★) indicates the optimum identified within the tested domain (40 °C, 1:4 *v*/*w*, 3% citric acid; Y = 5.41 ± 1.30%).

Temperature exerted the largest individual effect on yield (β_1_ = +0.729; *p* = 0.0017). Increased molecular mobility at 40 °C enhanced granule hydration and reduced interparticle cohesion in *S. tuberosum* L. granules below the gelatinization onset (Tg ≈ 56–70 °C) [17], facilitating starch release into the aqueous phase during centrifugation, consistent with the response surfaces shown in Figure 1. In the rejected-grade substrate, this effect was amplified because mechanical damage from the industrial rejection process (bruising, impact) had already partially disrupted cell membrane integrity, lowering the energy barrier for intracellular starch liberation.

The solid-to-liquid ratio (β_2_ = +0.695; *p* = 0.0025) showed comparable effect magnitude. The highest mean yields across conditions were obtained at a 1:3 ratio (2.07%), slightly above the 1:2 (1.79%) and 1:4 (1.66%) values when data were pooled over all acid concentrations. This reflects competing mechanisms: at 1:3, adequate dilution favors gravity-assisted sedimentation during centrifugation, while at 1:4 excess water disperses fine starch particles into the discarded supernatant [10]. Under the optimal combined conditions (T = 40 °C, R = 1:4, A = 3%), the synergistic T × A interaction more than compensates for these dilution-related losses, achieving the study’s maximum extraction yield.

The synergistic T × A interaction (β_13_ = +0.664; *p* = 0.028) reflects that, at 40 °C with 3% citric acid, partial protonation of cell-wall carboxylate groups weakens electrostatic interactions maintaining starch granules in a proteinaceous matrix. At lower temperatures this effect is insufficient to translate into measurable yield gains. The negative R × A interaction (β_23_ = −0.605; *p* = 0.043) indicates that high dilution combined with elevated acid concentration increases starch solubilization losses in discarded fractions, which is analogous to hydrolytic degradation of surface starch chains reported for citric acid above 3% in cassava starch systems [18].

### 3.3. Comparison Between Rejected and Standard-Grade Potato

Across the full experimental domain (n = 26 per material), rejected-grade potato yielded a significantly higher mean starch extraction than standard-grade material (3.056 ± 1.196% vs. 1.849 ± 0.534%; Welch’s t(34.6) = 4.699, *p* < 0.0001; Cohen’s d = 1.30, large effect). This population-level comparison, rather than a single-condition point estimate, establishes that the advantage of rejected-grade tubers is a robust and generalizable feature of the material, not an artifact of a particular operating condition.

At the process conditions identified as optimal for rejected-grade potato (40 °C, 1:4 *w*/*v*, 3% citric acid), rejected-grade material yielded 5.41 ± 1.30%, whereas standard-grade potato processed under the same conditions yielded only 1.49 ± 0.14% (3.6-fold difference); the full ranking of yields under selected conditions is presented in Table 3. Three complementary mechanisms explain this differential. First, mechanical damage from the industrial rejection process (impact, compression, and cutting) disrupts cell membrane integrity, pre-releasing starch granules into the intracellular aqueous phase and reducing extraction energy requirements relative to intact tubers. Second, the greater surface-to-volume ratio of smaller, irregularly shaped rejected tubers shortens the effective diffusion path for water and reagents during soaking. Third, enzymatic activity in stored damaged tubers may enhance granule dispersibility; this mechanistic hypothesis warrants direct testing in future work and is not established by the present data. Montoya-Anaya et al. [19] demonstrated that starch recovered from industrial crisp-fry residues retained full biochemical functionality despite its mechanical damage history, supporting the viability of rejected potato fractions as a functional starch source.

The higher biological variability of rejected material (CV = 39.1% vs. 28.9% for standard-grade potato, computed over the full dataset) reflects the compositionally heterogeneous nature of the reject stream. This variability is expected and does not compromise the optimization, since the industrial interest lies in processing variable feedstocks reproducibly under fixed conditions.

### 3.4. Enzymatic Quantification by Megazyme K-TSTA-100A (AOAC 996.11)

Two independent extractions performed under near-optimal conditions (40 °C, 1:4 *w*/*v*, 3% citric acid) yielded starch powders with moisture contents of 13.5% (Extraction 1) and 13.9% (Extraction 2) (AOAC 930.15). Reagent blank absorbances (A_510_ = 0.015) and sample blank absorbances (A_510_ = 0.022–0.025) were within the kit’s tolerance limits, confirming correct GOPOD reagent preparation and adequate extract clarification. Detailed quantification results are presented in Table 4.

The value of 83.4% d.b. is intermediate between the 74–77% d.b. reported for whole *S. tuberosum* L. tubers [8] and the 89% d.b. characteristic of commercially refined potato starch [20], a positioning consistent with laboratory-scale isolation without additional purification (Table 6). Sandoval-Aldana et al. [21] reported 85.87% carbohydrate content (d.b.) for starch isolated from the Leona Blanca variety by wet sedimentation, and Martínez et al. [22] obtained enzymatic-method purities of 89.7–92.2% d.b. for native Andean varieties, somewhat higher values reflecting the lower impurity load of intact native cultivars compared to mechanically damaged rejected material. These comparisons confirm the isolated starch meets quality standards suitable for biopolymer applications without intensive purification.

### 3.5. UV-Vis Spectrophotometric Confirmation of Starch Identity

The analytical performance of the UV-Vis iodine–starch complex method was evaluated through calibration with commercial soluble starch standards over the range 0–250 µg mL^−1^ at λ = 600 nm. Calibration data are presented in Table 5.

**Figure 2:**
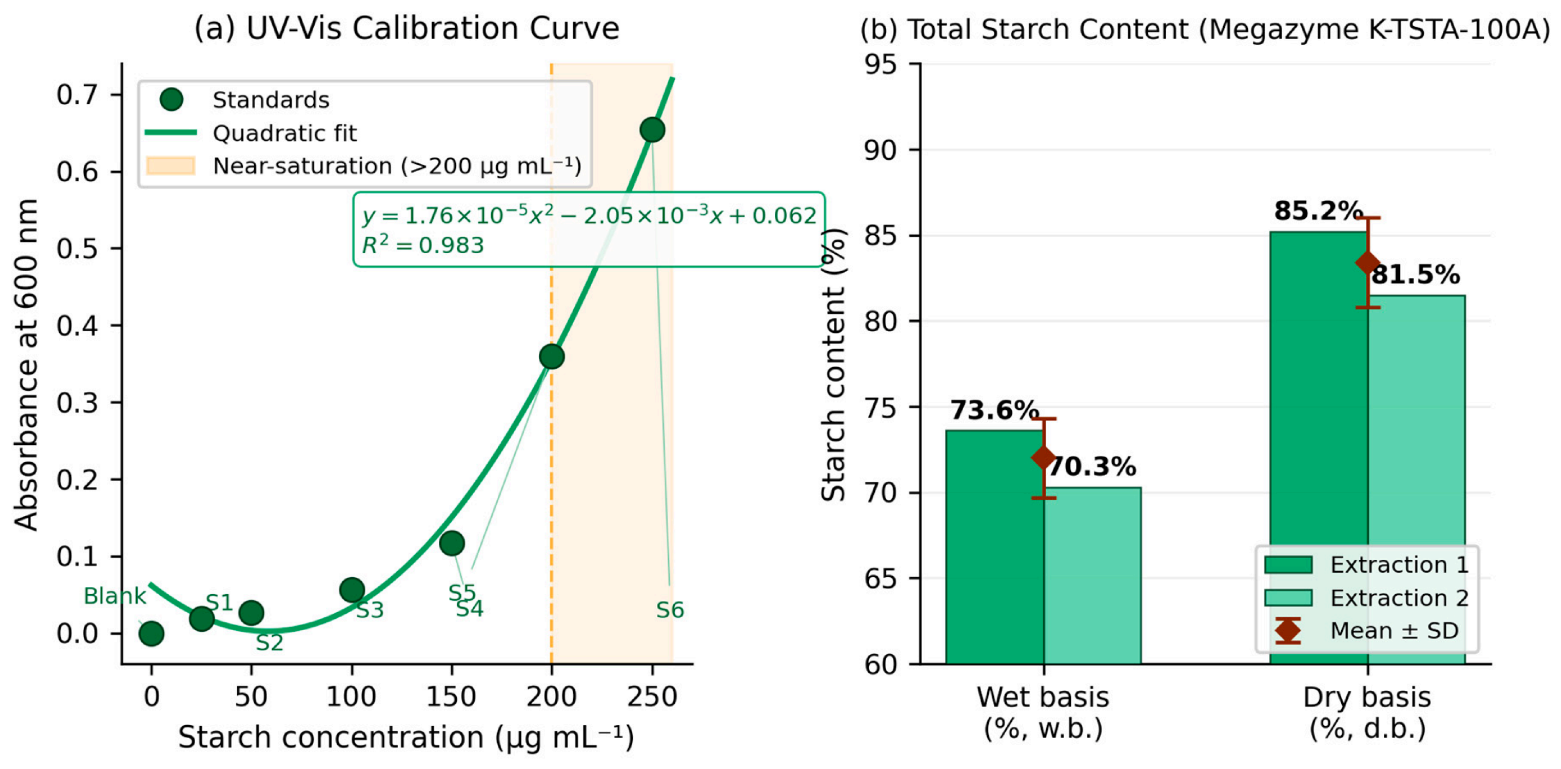
(**a**) UV-Vis calibration curve for the iodine–starch complex at λ = 600 nm (0–250 µg mL^−1^ commercial soluble starch standards); the quadratic fit (R^2^ = 0.9830) accounts for the non-linear response at higher concentrations; the shaded zone marks the near-saturation region (>200 µg mL^−1^); (**b**) total starch content by Megazyme K-TSTA-100A (AOAC 996.11) for two independent extractions on wet-weight (w.b.) and dry-weight (d.b.) bases; error bars = ±SD (n = 2 independent extractions); ◆ = global mean ± SD.

The calibration curve for the iodine–starch complex exhibited a quadratic response over the concentration range 0–250 µg mL^−1^ (y = 1.760 × 10^−5^x^2^ − 2.05 × 10^−3^x + 0.062; R^2^ = 0.9830; Table 5; Figure 2a). The departure from Beer–Lambert linearity above 150 µg mL^−1^ is well-documented for the I_2_/KI–amylose system and arises from progressive saturation of helical amylose binding sites, as the amylose:iodine molar ratio decreases at higher analyte concentrations [14]; the quadratic model adequately describes the full working range and was adopted for all standard-curve fitting.

Starch solutions prepared from the isolated powder (500 µg mL^−1^ nominal stock) produced a characteristic blue–violet colorimetric response upon reaction with Lugol’s reagent, with maximum absorbance at λ = 600 nm, confirming the presence of a well-formed amylose–triiodide inclusion complex [14,15]. The absorbance values obtained for sample aliquots were at the upper boundary of the calibration range, consistent with the high starch content previously established by the Megazyme enzymatic method (83.4 ± 2.6% d.b.). The iodine–starch complex was clearly distinguishable from the reagent blank and from the amylopectin-dominant signal, which absorbs primarily at 530–550 nm [15], confirming that the colorimetric response was dominated by the linear amylose fraction.

It should be noted that the absorbance values obtained for the isolated starch samples fell within the upper, non-linear portion of the calibration curve (>150 µg mL^−1^), a region where the iodine–starch complex approaches saturation. No attempt was made to back-calculate a quantitative starch concentration from this region, as doing so would extend the calibration beyond its validated linear range. The UV-Vis result is therefore restricted to confirmation of the characteristic colorimetric response of the iodine–starch complex (blue–violet color formation with λmax at 600 nm), and does not constitute an independent quantitative or structural confirmation of starch identity. Structural confirmation by FTIR or XRD is planned as part of a subsequent physicochemical characterization study within the PAPAPACK project.

The UV-Vis results are thus interpreted as confirmation of the characteristic iodine–starch colorimetric response in the extracted powder, consistent with native starch identity, complementing the enzymatic quantification (Section 3.4). Together, the two methods provide convergent analytical evidence: the Megazyme kit (AOAC 996.11) establishes the total starch content at 83.4 ± 2.6% d.b. with AOAC-validated precision, while UV-Vis spectrophotometry confirms the presence of an amylose-rich iodine-reactive fraction consistent with the reported 20–23% amylose content of native *S. tuberosum* L. starch [5,15]. This dual-method approach, with one quantitative (enzymatic) and one confirmatory (spectrophotometric) technique, constitutes a methodologically sound characterization strategy for agro-industrial starch fractions.

### 3.6. Comparative Context

To contextualize the present findings, Table 6 compares the yield and total starch content obtained in this work with values reported in the literature for potato industrial residues processed by related methods.

## 4. Conclusions

This study demonstrated that rejected *Solanum tuberosum* L. from industrial snack processing constitutes a superior starch source relative to standard-grade tubers under wet-process extraction. The Box–Behnken response surface design identified temperature (β_1_ = +0.729) and solid-to-liquid ratio (β_2_ = +0.695) as the dominant yield drivers, together with a significant synergistic temperature–acid interaction. Across the full experimental domain, rejected-grade potato yielded significantly more starch than standard-grade material (3.06 ± 1.20% vs. 1.85 ± 0.53%; Welch’s t = 4.70, *p* < 0.0001, Cohen’s d = 1.30), establishing this advantage as a robust population-level feature of the material rather than an isolated observation. Under the conditions identified as optimal within the tested domain (40 °C, 1:4 *w*/*v*, 3% citric acid), rejected-grade potato reached 5.41 ± 1.30%, a 3.6-fold increase over standard-grade material processed under the same conditions (1.49 ± 0.14%). Because this optimum lies at the boundary of the tested factor ranges, it should be interpreted as describing the best conditions identified within the explored domain rather than a confirmed global optimum; an expanded design extending temperature and solid-to-liquid ratio beyond the current limits is recommended before scaling to pilot operation. The yield advantage of rejected material is mechanistically attributable to pre-existing intracellular starch release from mechanical damage sustained during industrial rejection, which converts a waste stream into a preferred extraction substrate.

The isolated starch contained 83.4 ± 2.6% total starch on a dry-weight basis (Megazyme K-TSTA-100A, AOAC 996.11), with intra-extraction CV < 1.5% and a relative standard deviation between independent extractions below 5%. UV-Vis spectrophotometry (R^2^ = 0.9830) confirmed the presence of a native amylose-rich iodine-reactive fraction with characteristic absorption at 600 nm, consistent with the amylose content of native *S. tuberosum* L. starch. Suitability for biopolymer applications is inferred from starch purity and identity confirmation rather than demonstrated through direct functional testing; physicochemical characterization by SEM, FTIR, DSC, and XRD is identified as a necessary next step before industrial biopolymer applicability can be confirmed.

From a circular economy perspective, integrating agro-industrial reject streams into a starch supply chain for biodegradable packaging reduces both raw material costs and the environmental footprint of potato processing operations. The optimized conditions reported here are compatible with plant-level scale-up and provide a validated quantitative basis for subsequent work on physicochemical characterization (granule morphology by SEM, crystallinity by XRD, gelatinization thermodynamics by DSC, and functional group confirmation by FTIR), biopolymer film preparation, and life-cycle assessment of the valorization pathway.

## Figures and Tables

**Table 1:** Independent variables and coded levels in the Box–Behnken design.

Factor	Variable	Level −1	Level 0	Level +1	Coding
X_1_	Temperature (°C)	20	30	40	x_1_ = (T − 30)/10
X_2_	Solid:liquid ratio (*w*/*v*)	1:2	1:3	1:4	x_2_ = (R − 3)/1
X_3_	Citric acid (%, *w*/*v*)	0	3	6	x_3_ = (A − 3)/3

**Table 2:** ANOVA for the reduced Box–Behnken model: starch extraction yield from rejected *Solanum tuberosum* L.

Source	SS	df	MS	F	*p*-Value	SE	95% CI
Model (reduced)	22.677	4	5.669	9.236	0.0003 ***	—	—
x1—Temperature	8.52	1	8.52	11.92	0.0017 **	0.197	[0.318, 1.139]
x2—S:L ratio	7.74	1	7.74	10.83	0.0025 **	0.197	[0.285, 1.106]
x1x3—T × Acid	3.53	1	3.53	4.94	0.028 *	0.279	[0.083, 1.244]
x2x3—R × Acid	2.94	1	2.94	4.12	0.043 *	0.279	[−1.186, −0.024]
Residual	15.05	21	0.717	—	—	—	—
Lack of Fit	7.665	8	0.958	2.295	0.0882	—	—
Pure Error	5.427	13	0.417 (MS)	—	—	—	—
Total	41.45	25	—	—	—	—	—

Lack of Fit computed from the true replicate structure (13 unique conditions; pure error df = 13). SS: sum of squares; df: degrees of freedom; MS: mean square; SE: standard error of the coefficient; 95% CI: 95% confidence interval for significant terms. * *p* < 0.05; ** *p* < 0.01; *** *p* < 0.001.

**Table 3:** Ranked extraction yields under selected conditions for rejected *Solanum tuberosum* L. (n = 2 replicates per condition).

Conditions (T/R/A)	Ȳ (%)	SD (%)	Max (%)	Min (%)
40 °C/1:4/3%	5.41	1.30	6.32	4.50
40 °C/1:3/6%	4.04	0.87	4.87	3.21
30 °C/1:4/0%	3.95	0.64	4.42	3.48
40 °C/1:4/0%	3.79	0.55	4.19	3.39
30 °C/1:2/0%	1.25	0.34	1.52	0.98

Ȳ: mean yield; SD: standard deviation (n = 2). T: temperature; R: solid:liquid ratio; A: citric acid concentration.

**Table 4:** Total starch content by Megazyme K-TSTA-100A (AOAC 996.11) on isolated starch powder from rejected *Solanum tuberosum* L.

Parameter	Extraction 1	Extraction 2	Global Mean
Moisture (%, w.b.)	13.5	13.9	13.7 ± 0.3
Total starch (%, w.b.)	73.6	70.3	72.0 ± 2.3
Total starch (%, d.b.)	85.2	81.5	83.4 ± 2.6
Intra-extraction CV (%)	<1.5	<1.5	—
Reagent blank (A_510_)	0.015	0.015	—
Sample blank (A_510_)	0.022	0.025	—

w.b.: wet-weight basis; d.b.: dry-weight basis; CV: coefficient of variation; relative standard deviation between independent extractions < 5% (n = 2 independent extractions).

**Table 5:** Calibration data for iodine-reactive starch by UV-Vis spectrophotometry (λ = 600 nm).

Standard	Conc. (µg mL^−1^)	A_600_ (Corrected)	Regression ŷ
Blank	0	0.000	0.062
S1	25	0.019	0.022
S2	50	0.027	0.003
S3	100	0.056	0.033
S4	150	0.117	0.150
S5	200	0.360	0.356
S6	250	0.654	0.649

Calibration equation: y = 1.760 × 10^−5^x^2^ − 2.05 × 10^−3^x + 0.062; R^2^ = 0.9830. A_600_: absorbance at 600 nm against reagent blank.

**Table 6:** Literature comparison: starch extraction and total starch content from potato industrial residues.

Reference	Source Material	Method	Yield (%)	Starch d.b. (%)
This work	Rejected S. tuberosum L. (CO)	BBD-RSM wet proc.	5.41 (opt.)	83.4 ± 2.6
Montoya-Anaya et al. [19]	Crisp-fry residue (MX)	Wet sedimentation	—	~80
Sandoval-Aldana et al. [21]	Leona Blanca (CO)	Wet sedimentation	—	85.9
Martínez et al. [22]	Andean native vars. (PE)	Enzymatic	—	89.7–92.2
Brążkiewicz et al. [8]	Commercial vars. (PL)	Lab isolation	—	74.0–77.6
Xu et al. [20]	Commercial refined (CN)	Industrial	—	89.3

—: not reported; d.b.: dry-weight basis; CO: Colombia; MX: Mexico; PE: Peru; PL: Poland; CN: China.

## Data Availability

The original contributions presented in this study are included in the article/Supplementary Materials. Further inquiries can be directed to the corresponding author.

## Supplementary Materials

The following supporting information can be downloaded at: https://www.mdpi.com/article/10.3390/ma19153197/s1. The Box–Behnken design matrix with complete experimental data for both raw materials and raw spectrophotometric calibration data are available as Supplementary Materials at the MDPI web page of this article: Table S1: Complete Box-Behnken design matrix and raw starch extraction yield data for rejected-grade and standard-grade potato (26 observations per material); Table S2: Raw UV-Vis calibration absorbance data (Standards S1–S6 and blank).

## Abbreviations

The following abbreviations are used in this manuscript:

**Abbreviation**	**Definition**
BBD	Box–Behnken Design
RSM	Response Surface Methodology
ANOVA	Analysis of Variance
AMG	Amyloglucosidase
GOPOD	Glucose Oxidase–Peroxidase Reagent
LoF	Lack of Fit
CV	Coefficient of Variation
d.b.	Dry-weight Basis
w.b.	Wet-weight Basis
AOAC	Association of Official Analytical Chemists
AACC	American Association of Cereal Chemists
ICC	International Association for Cereal Science and Technology
