# Supplementary material for: Starch Extraction from Rejected Solanum tuberosum L. Optimized by Box–Behnken Design: Enzymatic and UV-Vis Spectrophotometric Quantification
Source: Materials (Basel). 2026 Jul 27;19(15):3197. doi: 10.3390/ma19153197 (PMC13466238; doi:10.3390/ma19153197)
Supplement: Supplementary file 1 [file materials-19-03197-s001.zip › materials-4385355-supplementary.pdf]

Supplementary Materials

materials-4385355 Starch Extraction from Rejected Solanum tuberosum L. Optimized by Box–Behnken Design: Enzymatic and UV-Vis Spectrophotometric Quantification

Table S1. Complete Box–Behnken design matrix and raw starch extraction yield data

Raw yield values (%, dry basis) for all 26 observations per material (13 unique experimental conditions × 2 biological replicates).

Material	Replicate	Temperature (°C)	S:L Ratio	Citric Acid (%)	Yield (%, d.b.)
**Rejected-grade**	1	40	3	6	3.424
**Rejected-grade**	2	40	3	6	4.652
**Rejected-grade**	1	20	3	6	1.976
**Rejected-grade**	2	20	3	6	2.569
**Rejected-grade**	1	40	2	3	3.874
**Rejected-grade**	2	40	2	3	3.977
**Rejected-grade**	1	20	4	3	3.486
**Rejected-grade**	2	20	4	3	1.979
**Rejected-grade**	1	20	2	3	1.600
**Rejected-grade**	2	20	2	3	1.694
**Rejected-grade**	1	30	4	0	3.596
**Rejected-grade**	2	30	4	0	4.306
**Rejected-grade**	1	30	3	3	3.366
**Rejected-grade**	2	30	3	3	2.029
**Rejected-grade**	1	30	2	6	3.027
**Rejected-grade**	2	30	2	6	3.320
**Rejected-grade**	1	40	3	0	1.752
**Rejected-grade**	2	40	3	0	2.539
**Rejected-grade**	1	30	4	6	3.383
**Rejected-**	2	30	4	6	3.536

**grade**					
**Rejected-grade**	1	20	3	0	3.099
**Rejected-grade**	2	20	3	0	2.972
**Rejected-grade**	1	30	2	0	1.523
**Rejected-grade**	2	30	2	0	0.967
**Rejected-grade**	1	40	4	3	4.492
**Rejected-grade**	2	40	4	3	6.324
**Standard-grade**	1	40	3	6	1.389
**Standard-grade**	2	40	3	6	1.789
**Standard-grade**	1	20	3	6	1.288
**Standard-grade**	2	20	3	6	1.990
**Standard-grade**	1	40	2	3	1.486
**Standard-grade**	2	40	2	3	1.787
**Standard-grade**	1	20	4	3	1.983
**Standard-grade**	2	20	4	3	1.295
**Standard-grade**	1	20	2	3	1.391
**Standard-grade**	2	20	2	3	0.992
**Standard-grade**	1	30	4	0	2.098
**Standard-grade**	2	30	4	0	1.288
**Standard-grade**	1	30	3	3	1.799
**Standard-grade**	2	30	3	3	1.897
**Standard-grade**	1	30	2	6	1.988
**Standard-grade**	2	30	2	6	1.497
**Standard-grade**	1	40	3	0	2.590
**Standard-grade**	2	40	3	0	3.085
**Standard-grade**	1	30	4	6	1.590
**Standard-grade**	2	30	4	6	1.791

**Standard-grade**	1	20	3	0	2.891
**Standard-grade**	2	20	3	0	1.998
**Standard-grade**	1	30	2	0	2.597
**Standard-grade**	2	30	2	0	2.597
**Standard-grade**	1	40	4	3	1.589
**Standard-grade**	2	40	4	3	1.390

Table S2. Raw UV-Vis calibration absorbance data

Absorbance readings at $\lambda = 600$ nm for the iodine-starch complex calibration curve, commercial soluble starch standards (Merck KGaA).

Standard	Concentration (μg mL^{-1})	Absorbance at 600 nm (corrected)
**Blank**	0	0.000
**S1**	25	0.019
**S2**	50	0.027
**S3**	100	0.056
**S4**	150	0.117
**S5**	200	0.360
**S6**	250	0.654

Calibration equation: $y = 1.760 \times 10^{-5}x^2 - 2.05 \times 10^{-3}x + 0.062$; $R^2 = 0.9830$. Quadratic fit accounts for departure from Beer–Lambert linearity above 150 μg mL^{-1}, consistent with progressive saturation of helical amylose binding sites.
